# Risk factors for cardiovascular adverse events from immune checkpoint inhibitors

**DOI:** 10.3389/fonc.2023.1104888

**Published:** 2023-04-28

**Authors:** Lingli Luo, Yuxin Liu, Jingfen Lu, Yifei Zhang, Gang Fan, Xiaojun Tang, Weiming Guo

**Affiliations:** ^1^ Medical College, Hunan Polytechnic of Environment and Biology, Hengyang, China; ^2^ College of Traditional Chinese Medicine, Hunan University of Chinese Medicine, Changsha, China; ^3^ The First Clinical Medical College, Guangzhou University of Chinese Medicine, Guangzhou, China; ^4^ The First Teaching Hospital, Tianjin University of Traditional Chinese Medicine, Tianjin, China; ^5^ Urology Department, Huazhong University of Science and Technology Union Shenzhen Hospital, the 6th Affiliated Hospital of Shenzhen University Health Science Center, Shenzhen, China; ^6^ Department of Spinal Surgery, The Second Affiliated Hospital, Hengyang Medical School, University of South China, Hengyang, China; ^7^ Sports Medicine Department, Huazhong University of Science and Technology Union Shenzhen Hospital, the 6th Affiliated Hospital of Shenzhen University Health Science Center, Shenzhen, China

**Keywords:** immune-related cardiovascular adverse events, immune checkpoint inhibitor, immunomodulation, cardiovascular adverse events, risk factors, systemic immunity

## Abstract

Immune-related adverse events (irAEs), including skin injury, liver and kidney injury, colitis, as well as cardiovascular adverse events, are a series of complications arising during the treatment of immune checkpoint inhibitors (ICIs). Cardiovascular events are the most urgent and the most critical, as they can end life in a short period of time. With the widespread use of ICIs, the number of immune-related cardiovascular adverse events (irACEs) induced by ICIs has increased. More attention has been paid to irACEs, especially regarding cardiotoxicity, the pathogenic mechanism, diagnosis and treatment. This review aims to assess the risk factors for irACEs, to raise awareness and help with the risk assessment of irACEs at an early stage.

## 1 Introduction

The application of immune checkpoint inhibitors (ICIs) has brought new breakthroughs and hope for the treatment of cancer (1). However, ICI use also results in skin injury, liver and kidney injury, cardiovascular events, colitis and other adverse reactions, among which immune-related cardiovascular adverse events (irACEs) are the most serious adverse events. irACEs can include but are not limited to myocarditis, arrhythmia, pericardial effusion, heart failure, atrial fibrillation, myocardial infarction and cardiac arrest (2). According to the World Health Organization database, myocarditis and arrhythmia, with high fatality rates, are the most common irACEs (3). A National Cancer Institute study showed that, out of 6,925 patients, 40 (0.6%) received irACEs and 18 (45%) died, 4 of whom had concurrent myositis (22.5%) (4). Salem et al. reported that mortality from myocarditis is as high as 50% (5). Ekaterina et al. reported that a patient with previous hypertension and moderate left ventricular dysfunction died from severe adverse effects after the first infusion of atezolizumab, with a histopathological diagnosis of myocarditis, tumour coronary artery embolization, and micrometastases of myocardial mucus carcinoma; high expression of PD-1 in the myocardial area without tumour cells was also detected (6). Although the cause of death cannot be completely attributed to ICIs, judging from the histopathological diagnosis and the high detection results of PD-L1, the use of atezolizumab aggravated the patient’s diagnosis and contribute to death.

As ICIs are more and more widely used, they also cause more and more irACEs, which has attracted considerable attention and vigilance (7). However, irACEs may occur with irAEs in other systems, basic cardiovascular disease and/or the superposition of tumour disease symptoms. There are no specific clinical signs and symptoms or specific indicators to make many clinicians reconsider prescribing ICIs, combined with poor cardiovascular risk awareness. This results in making the early detection of irACEs difficult, leading to serious consequences. Therefore, this mini-review aims to summarize and elaborate the risk factors for irACEs in patients, to improve the cardiovascular risk awareness of clinicians regarding the use of ICIs and to advocate for the comprehensive identification and assessment of risk for the purpose of reducing the occurrence of irACEs and death.

## 2 Risk factors

It is currently known that the risk factors related to patients treated with irACEs are patient-related risk factors and treatment-related risk factors (8). Clinicians need to evaluate the relevant risk factors before medication, to determine whether patients are suitable for using ICIs drugs and fully inform the patient of related risks and benefits according to the condition and risk factors personalized medication plan, followed by a reasonable choice of drug type and dosage. **Table 1** example shows the guidelines and the published cardiac risk factors (9–12).

**Table 1:** Risk factors for the treatment of immune checkpoint inhibitors.

AHA-2020	ESMO-2020	ECS-2022	ASCO-2017
High-dose anthracycline (eg, doxorubicin ≥250 mg/m^2^, epirubicin ≥600 mg/m^2^) •High-dose radiotherapy (≥30 Gy) where the heart is in the treatment field •Lower-dose anthracycline (eg, doxorubicin <250 mg/m^2^,epirubicin <600mg/m^2^) or HERis or VEGFis or proteasomeis or Bcr-Ablis and presence of any of the following factors: ∘Age ≥60 y ∘Lower-dose radiotherapy (<30 Gy) where the heart is in the treatment field ∘≥2 Risk factors, includingsmoking, hypertension, diabetes mellitus, dyslipidemia, chronic renal insufficiency, and obesity •Previous heart disease •Elevated cardiac biomarkers* before initiation of anticancer therapy	•Prior anthracycline-based treatment •>75 years old or <10 years old •Prior mediastinal or chest radiotherapy •Hypertension (before or at the time of treatment) •Smoking exposure (current or previous) •Previous combined treatment with trastuzumab and an anthracycline •Elevated cardiac biomarkers before initiation of anticancer therapy •Baseline abnormal systolic left ventricular function with LVEF<50% •Pre-existing diabetes mellitus	**Clinical factors** •Pre-existing cardiovascular disease •ICI combination •Early first symptoms/signs after ICI initiation •Cardiac arrest •Heart failure and cardiogenic shock •Oxygen dependence •Low diastolic blood pressure •Simultaneous non-cardiovascular irAE, especially myositis and myasthenia gravis **Electrocardiogram** •Severe conduction disorders and ventricular arrhythmias •QRS ≥100 ms •Decrease in QRS voltage **Echocardiography** •Low |GLS| in echocardiography |GLS| < 13% if LVEF <50% |GLS| < 16% if LVEF ≥50% **Cardiac magnetic resonance imaging** •Myocardial native T1 value on CMR T1 >mean value ±2 standard deviations of the site norm •Septal LGE on CMR **Serum biomarkers** •Troponin High Troponin T values at admission/peak/discharge Discharge Troponin T value ≥1.5 ng/mL Admission Troponin I value ≥3.73 ng/mL •Absolute lymphocyte count decrease ≥35% to admission •Neutrophil/lymphocyte ratio increase ≥100% to admission **Endomyocardial biopsy** •Degree of lymphocyte infiltration on EMB >50 CD3+ cells/high-power field **Management** •Delay in initiation of high-dose corticosteroids •Requirement of intensified immunosuppressive therapy	High-dose anthracycline (eg, doxorubicin ≥250 mg/m^2^, epirubicin ≥600 mg/m^2^) •High-dose radiotherapy (≥30 Gy) where the heart is in the treatment field •Lower-dose anthracycline (eg, doxorubicin <250mg/m^2^, epirubicin <600mg/m^2^) in combination with lower-dose RT (<30 Gy) •Treatment with lower-dose anthracycline (doxorubicin <250 mg/m^2^, epirubicin <600 mg/m^2^) or trastuzumab alone, and presence of any of the following risk factors: ∘Multiple cardiovascular risk factors (≥ two risk factors), including smoking, hypertension, diabetes, dyslipidemia, and obesity, during or after completion of therapy. ∘Older age (≥ 60 years old) at cancer treatment ∘Compromised cardiac function (eg, borderline low LVEF [50% to 55%], history of myocardial infarction, ≥ moderate valvular heart disease) at any time before or during treatment •Treatment with lower-dose anthracycline (eg, doxorubicin <250 mg/m^2^, epirubicin <600 mg/m^2^) followed by trastuzumab (sequential therapy)

HERis, human epidermal growth factor-2 inhibitors; VEGFis, vascular endothelial growth factor inhibitors; proteasomeis, proteasome inhibitors; Bcr-Ablis , Bcr-Abl kinase inhibitors; *N-terminal pro-B-type natriuretic peptide (or B-type natriuretic peptide) and/or troponin. CMR, cardiac magnetic resonance; EMB, endomyocardial biopsy; ICI, immune checkpoint inhibitor; irAE, immune-related adverse event; |GLS|, global longitudinal strain absolute value; LGE, late gadolinium enhancement; LVEF, left ventricular ejection fraction.

### 2.1 Individual factors

Patient-related risk factors mainly include age, gender, BMI and smoking history, as well as the history of cardiovascular disease and combined diseases (e.g., dyslipidaemia, hypertension and diabetes mellitus), type of malignancy, underlying autoimmune disease, opportunistic pathogen infection and genetic susceptibility (13). In a report provided by Kalinich et al., severe irAE rates were higher in males at in 59.1% (n=298), compared to the female population at 40.9% (n=206). This phenomenon lacks an explanation (14). However, more irAEs have been reported in women, which may be related to their vulnerability to autoimmune diseases, including systemic lupus erythematosus (SLE), multiple sclerosis (MS) and rheumatoid arthritis (15–17). A high Body Mass Index(BMI) is a risk factor for the development of irACEs, and studies have shown a 9% increase in irACEs per 1kg/m^2^ increase in BMI (18). Another study suggested that age over 65 years, the presence of metastatic disease or hypertension, and a platelet to lymphocyte ratio <180 are risk factors for irACEs (19). Patients with a significant history of heart disease and uncontrolled hypertension, and those who have been treated with HER2 inhibitors, VEGF TKIs and anthracyclines are at a very high risk, and responses to ICIs should be closely and continuously observed (13). The irACEs incidence rate was also closely related to the type of primary tumor. Melanoma (37.5%) and gastric cancer (12.5%) were both primary tumor with high incidence regardless of whether PD-(L)1 was used alone or in combination with other ICIs,while the rate of gynecologic cancer was 7.5% and lung cancer and lymphoma were both 5% (4). Therefore, before using ICIs, patients should focus on whether the above risk factors exist and conduct a risk assessment.

### 2.2 Treatment-related factors

Treatment-related risk factors include the use of ICI medication type, dose, and combination therapy (e.g., radiotherapy, chemotherapy, targeted therapy and other ICIs, or immunotherapy). Drug type and dose are closely related to irACEs. In terms of drug type, anti-PD-1/PD-L1 and anti-CTLA-4 monotherapy cause irACEs such as myocarditis; anti-PD-1/PD-L1 treatment causes more related pericardial diseases, and more temporal arteritis is caused by anti-CTLA-4 monotherapy (5). In terms of drug dose, a meta-analysis by Bertrand et al. suggested that the dose was associated with the risk of developing all irACEs, with an incidence of 61% when the ipilimumab dose was 3 mg/kg and 79% with increasing the ipilimumab dose to 10 mg/kg (20). Another meta-analysis that compared the case fatality rate with monotherapy between 3 mg/kg ipilimumab (1,438 patients) and 10 mg/kg ipilimumab (3,016 patients) found a higher fatality rate with 10 mg/kg (2).

Combination therapy, especially the combination of dual ICIs, has been reported to be effective in overcoming cancer (21). However, there are relevant research tables showing that the combination of two or more ICIs increases the risk of irACEs. According to the WHO database, the combination of ICIs resulted in a nearly two-fold increase in irACE-related myocarditis mortality rates (67% vs. 36%), as compared with patients receiving anti-PD-L1 monotherapy (7). A meta-analysis showed that PD-1/PD-L1 inhibitor combined with chemotherapy increased the risk of full-grade arrhythmia and hypertension; especially a PD-L1 inhibitor combined with chemotherapy caused more arrhythmias (22).

In addition, the combination of radiotherapy and ICIs also increases the risk of irACEs, such as the combination of chest radiation and PD-1 inhibitors that can aggravate radiation-induced cardiac inflammation and cardiotoxicity (23). Prevention of opportunistic infections and influenza vaccination is important to reduce the risk of irACEs occurrence. A study by Awadalla found that, in myocarditis cases, patients receiving influenza vaccination 6 months before ICIs or during ICI treatment had a lower incidence of myocardial infarction and a lower incidence of irACEs (36% vs.55%, p=0.10); cumulative irACEs in naïve patients was more than twice that of FV patients (24).

## 3 Potential mechanism

Although there have been many basic studies on irACEs, the mechanism is very complex and still undefined; however, the main reason recognized by most researchers is the overactivation of T cells.

**Figure 1:**
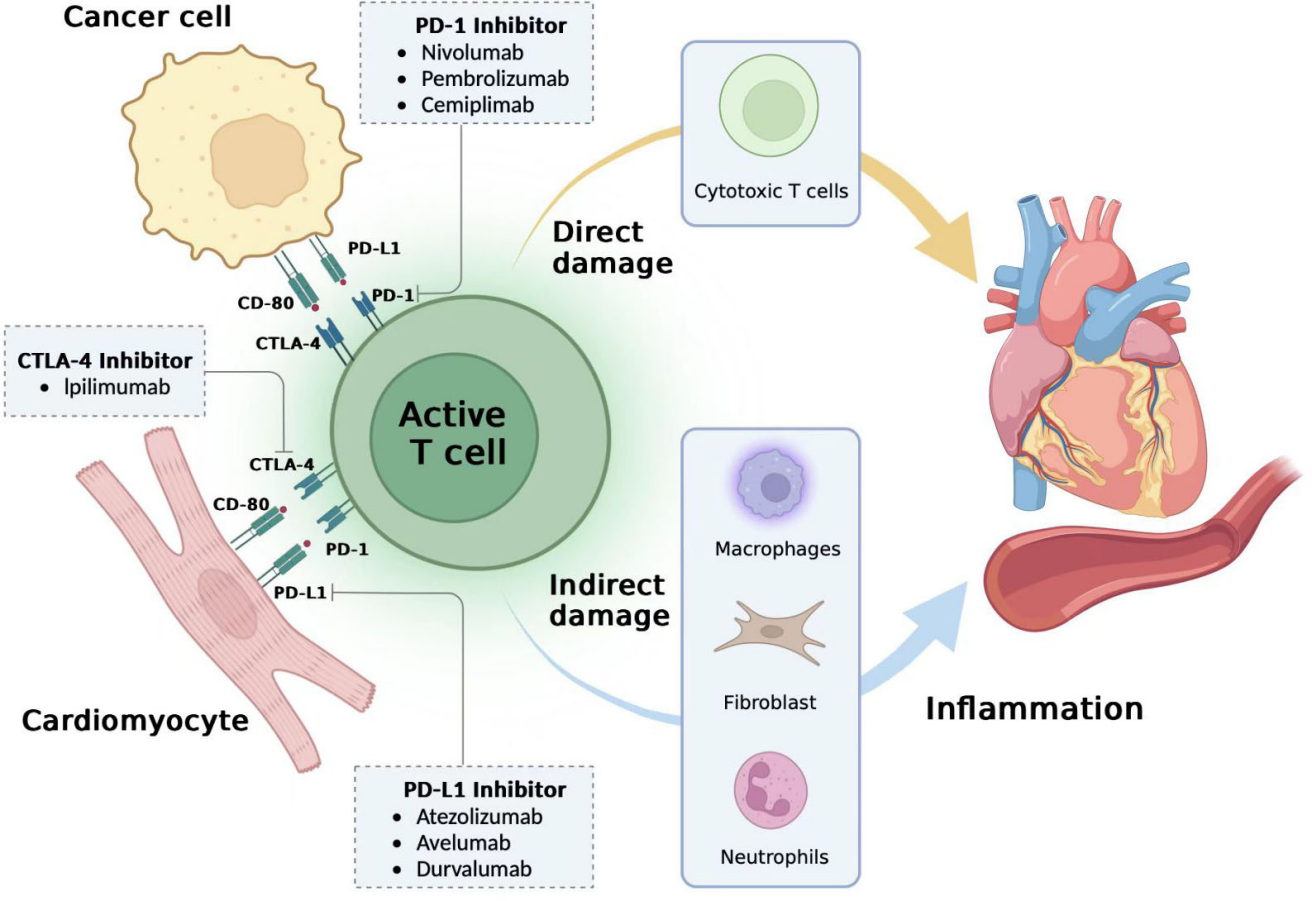
Potential mechanisms of immune-related cardiovascular adverse events. The mechanism of immune-related adverse cardiovascular adverse events (irACEs) is unclear, and it has been proposed that overactivation of T cells is the main cause. Cardiomyocytes may have a co-antigen progra m-med death-ligand 1 (PD-L1) or CD-80 on the surface of tumor cells, and they can normally bind to programmed death 1 (PD-1) or cytotoxic T-lymphocyte-associated antigen 4 (CTLA-4) on the T cell surface to prevent excessive activation of T cells. However, after the use of immune checkpoint inhibitors (ICIs) such as PD-1 inhibitors, PD-L1 inhibitors and CTLA-4 inhibitors, cardiomyocytes and tumor cells, CTLA-4/CD-80 and T cells are hyperactivated, T cells through cytotoxic T cells directly damage cardiovascular tissue, and recruit monocytes, macrophages, neutrophils and other inflammatory response, indirectly damage the cardiovascular system and finally cause irACEs.irACEs, immune-related cardiov-ascular adverse events; PD-1, programmed death 1; PD-L1, programmed death-ligand 1; CTLA-4, cytotoxic T-lymphocyte-associ ated antigen 4; ICIs, Immune Checkpoint Inhibitors. Figures generated with BioRender (https://biorender.com/).

The pathology of myocarditis in irACEs is characterized by plaque infiltration of macrophages as well as CD4+ and CD8+ T lymphocytes in the myocardium and the conduction system (including the atrioventricular node), along with cardiomyocyte death; B cells are rarely seen (25). The reason for the infiltration of T cells into myocardial tissue may be the cross-reaction of antigens of myocardial tissue and antigens of tumour tissue, which enables T cells, macrophages and monocytes to infiltrate into normal myocardial tissue after being activated (26). Recent studies suggest that the clinically important autoantigen in ICI myocarditis may be the cardiac-specific protein alpha-myosin, which is asthe cognate antigen source for three major histocompatibility complex class I-restricted T cell receptors derived from mice with fulminant myocarditis (27). After treatment with ICIs, T cells are activated and proliferate, and pro-inflammatory cytokines such as IL-1α, IL-2, IFNα2 and IL-17 indirectly damage myocardial cells; meanwhile, cytotoxic T cells directly kill myoblasts, fibroblasts, endothelial cells and mesothelial cells, causing myocarditis (28–30) (**Figure 1**).

Programmed death 1 (PD-1) and programmed death-ligand 1 (PD-L1) are considered to be the common antigen between cardiomyocytes and tumour tissue. PD-1 binds to PD-L1 under normal circumstances to inhibit the abnormal activation of T cells and prevent the occurrence of immune myocarditis. However, after the use of a PD-1/PD-L1 inhibitor, PD-L1 fails to bind normally to PD1, thereby over activating T cells. Cardiomyocytes lack a protective mechanism, causing direct or (and) indirect damage leading to cardiovascular tissue damage and irACEs (31) (**Figure 1**). On the other hand, PD-L1 can reflect the extent of cardiomyocyte damage to the immune system. In a study on lymphocytic myocarditis and ICIs, PD-L1 in normal myocardial was very low at baseline, but PD-L1 in lymphocytic myocarditis (30), PD-L1 in MI (25), and PD-L1 expression after cardiac ischemia-reperfusion injury (32). Thus, irACEs production is closely correlated with the blockade of PD-1/PD-L1. In addition, cytotoxic T-lymphocyte-associated antigen 4 (CTLA-4) has been shown to have a similar effect. CTLA-4 inhibitors reduce the inhibition of CTLA-4, leading to the accumulation of T cells and increased T cell activity in the cardiac environment, leading to cardiac damage (33).

## 4 Discussion

The high incidence of cancer has led to ICI use by more people, so the number of irACE-related deaths is increasing. Cardiology and oncology professional associations have issued statements that patients receiving potential cardiovascular toxic cancer treatment should be subjected to a baseline cardiovascular disease risk assessment (34), to determine risk factors before ICIs and careful medication. Previously, we briefly explained the patient-related risk factors and treatment-related risk factors and mechanisms, mainly because underlying cardiovascular disease, high-dose drug use and combination therapy increase the risk of irACEs. We cannot change and avoid patient-related risk factors, such as gender, age, BMI, smoking history, and underlying diseases, but we can identify high-risk patients through these factors, strengthen management, and try to reduce the risk before treatment. For example, patients with hyperglycaemia, hyperlipidaemia and hypertension are more likely to experience adverse cardiovascular events; the use of ICIs increases this risk, so before treatment, treatment and after treatment patients should adjust their diet and regular medication, seek the advice of professional cardiovascular doctors to assist with treatment, and maintain strict control of blood sugar, blood lipids and blood pressure to ensure that ICI treatment under relatively safe conditions. However, these identified risk factors are only based only on limited retrospective studies, and more prospective trials are needed to clarify additional risk factors.

Treatment risk also needs to be controlled, and the dose and regimen choice are difficult to balance with treatment risk. When a patient’s tumour disease progresses, it is often necessary to increase the dose of ICIs or combine them with other anti-tumour treatments. Therefore, how to skilfully achieve the anti-tumour effect and avoid irACEs needs the support of rich clinical experience and reliable evidence-based medical evidence. A mathematical model has been proposed to simulate the immune system and calculate the optimal medication regimen based on the patient’s condition to minimize the risk of treatment and avoid irACEs (35). However, none of these models fully considers the various types of immune cells, cytokines and their interactions of, and further clinical trials are needed to demonstrate the reliability of the mathematical models. There are no specific drugs for irACEs, but guidelines generally recommend high-dose corticosteroids (12mg/kg) for the treatment of irACEs, especially myocarditis (12, 36). However, in some patients corticosteroid therapy is ineffective, but the use of other immunomodulatory drugs can significantly relieve the symptoms, such as tofacitinib and intravenous immunoglobulins (IVIG) (37, 38). Therefore, the treatment of irACEs with immunomodulatory drugs deserves in-depth study and development.

When risks are unavoidable, they should be diagnosed and treated as early as possible. Test indicators and imaging tests of minimal trauma are more easily accepted and applied clinically, and the predictive specificity indicators of cardiac immunity, such as cytokines and microRNAs, deserve intensive research and development (27, 39, 40).

In cases where the mechanism is not clear, and no specific drugs are available to reduce the occurrence and deaths related to irACEs, doctors should comprehensively assess patient risk factors before the use of ICIs, evaluate medication risk, choose the best treatment, and be alert to irACEs through various means of early detection and diagnosis according to the guidelines grading management and treatment, until several years after the use of these medications. Several guidelines from different organizations, including the European Society of Medical Oncology (ESMO), Society of Clinical Oncology (ASCO), the National Comprehensive Cancer Network (NCCN), the American and the Society for Cancer Immunotherapy (SITC), have been published to inform clinicians on the diagnosis and management of ICIs (10, 41–43). More prospective clinical studies and evidence-based medical evidence are needed to help physicians identify risk factors for the early diagnosis and treatment of irACEs. In terms of new drug development, it is necessary to develop new immunomodulatory drugs according to the discovered mechanisms to avoid excessive activation of T cells in heart tissue, and to explore other irACE pathogenic mechanisms to develop non-cardiotoxic ICIs or immunomodulatory drugs.

## Author contributions

All authors listed have made a substantial, direct, and intellectual contribution to the work and approved it for publication.
